# Novel Applications of Non-Invasive Intravesical Botulinum Toxin a Delivery in the Treatment of Functional Bladder Disorders

**DOI:** 10.3390/toxins13050359

**Published:** 2021-05-18

**Authors:** Jia-Fong Jhang, Hann-Chorng Kuo

**Affiliations:** Department of Urology, Buddhist Tzu Chi General Hospital, 707 Chung-Yang Road, Section 3, Hualien 970, Taiwan; alur1984@hotmail.com

**Keywords:** botulinum toxin, transport, deliver, shock wave

## Abstract

Although intravesical botulinum toxin type A (BoNT-A) injection for functional bladder disorders is effective, the injection-related problems—such as bladder pain and urinary tract infection—make the procedure invasive and inconvenient. Several vehicles have recently been developed to deliver BoNT-A without injection, thereby making the treatment less or non-invasive. Laboratory evidence revealed that liposome can carry BoNT-A across the uroepithelium and act on sub-urothelial nerve endings. A randomized placebo controlled study revealed that intravesical administration of liposome-encapsulated BoNT-A and TC-3 hydrogel embedded BoNT-A can improve urinary frequency, urgency, and reduce incontinence in patients with overactive bladders. A single-arm prospective study also revealed that intravesical administration of TC-3 hydrogel embedded BoNT-A can relieve bladder pain in patients with interstitial cystitis/bladder pain syndrome (IC/BPS). We recently administered suprapubic energy shock wave (ESW) after BoNT-A intravesical administration in six patients with IC/BPS. Although pain reduction and symptom improvement were not significant, immunochemical staining showed cleaved synaptosome-associated protein 25 in the bladder after the procedure. This suggests that ESW can promote passage of BoNT-A across the uroepithelium. In conclusion, using vehicles to intra-vesically deliver BoNT-A for functional bladder disorders is promising. Further studies are necessary to confirm the efficacy and explore novel applications.

## 1. Introduction

Functional bladder disorders, such as overactive bladder (OAB) and interstitial cystitis/bladder pain syndrome (IC/BPS) are common among men and women of all ages [1]. The estimated prevalence of OAB and IC/BPS is approximately 10.7% and 4.2%, respectively [2,3]. The quality of life in patients with functional bladder disorders may be negatively impacted by urinary frequency, urgency, incontinence, and bladder pain [1]. Although anti-muscarinics and β_3_ adrenergic agonists can relieve symptoms in patients with OAB, overall patient satisfaction rate was approximately 55–75% [4]. Effective oral medication options for IC/BPS are limited, and most patients with IC/BPS require long-term pain control medications [5]. Hence, intravesical medications have been developed for patients with refractory functional bladder disorders. Using intravesical capsaicin and resiniferatoxin for patients with refractory OAB can significantly decrease urinary frequency and incontinence; however, almost 50% of patients suffered from acute suprapubic pain [6]. Intravesical administration of hyaluronic acid can relieve bladder pain in patients with IC/BPS, but there may not be significant improvement in urinary frequency [7,8]. Clinicians continue to seek more effective intravesical medications with fewer side effects for patients with refractory functional bladder disorders.

Botulinum toxin (BoNT) is a potent neurotoxic protein that is produced by *Clostridium botulinum*. BoNT type A (BoNT-A) plays an important role in the management of a wide variety of diseases [9]. Regarding lower urinary tract diseases, BoNT-A injection was first introduced into the urethral sphincter in patients with spinal cord injury and detrusor–sphincter dyssynergia in 1988 [10]. Intravesical BoNT-A injection is now widely used to treat patients with functional bladder disorders, and can also improve urinary frequency, incontinence, and bladder pain in patients with refractory OAB and IC/BPS [11,12]. It is currently listed as a standard treatment in the clinical guidelines for OAB and IC/BPS [5,13]. However, some critical issues of intravesical BoNT-A injection still need to be resolved in clinical practice. First, intravesical injection might result in an approximately 5% risk of bothersome complications, such as hematuria and urinary tract infection [11,12]. Second, bladder pain during the procedure can be severe and unbearable in some patients. General anesthesia makes intravesical injection tolerable, but attracts an additional cost and inconvenience. Additionally, the efficacy of BoNT-A declines at six months after the injection, and a repeat injection might be necessary when symptoms recur [11,12]. Hence, urologists require a more convenient and less invasive intravesical BoNT-A delivery method for patients with functional bladder disorders. The inner lining of the urinary bladder is a specialized stratified epithelium (urothelium) which serves as a barrier to ions, chemicals, and microbes in urine [14]. Some novel vehicles have recently been developed to carry BoNT-A across the urothelium, and their efficacy in treating functional bladder disorders has been proven in clinical studies. This article aims to review recent advances in novel vehicles to promote BoNT-A delivery in patients with functional bladder disorders, and to present our recent experience of using extracorporeal energy shock wave to enhance intravesical BoNT-A administration.

## 2. The Mechanism of Bont-A in Functional Bladder Disorders

As a potent neurotoxin, BoNT inhibits neurotransmitter acetylcholine release from efferent nerves at neuromuscular junctions [9]. In the presynaptic space, BoNT enters the neuronal cell via receptor-mediated endocytosis [15]. BoNT subsequently separates into 50 KDa light chain and 100 KDa heavy chain in the endosomal vesicle [9]. The light chain of BoNT is the biologically active moiety. It can cleave synaptosome-associated protein 25 (SNAP-25) in the presynaptic nerve, which accounts for membrane fusion by forming a tight complex of synaptic vesicle and cell membranes. The cleaved SNAP-25 cannot mediate fusion of vesicles with the cell membrane in the nerve terminal membrane, thus inhibiting the release of the neurotransmitter from axon endings, and ultimately causing flaccid paralysis of muscles [9]. Initially, the effects of BoNT-A in treating functional bladder disorders were expected to be attributed to the effect of detrusor muscle paralysis. However, recent evidence revealed that BoNT-A injection into the bladder can also modulate sensory function by blocking various noxious neurotransmitter release, including adenosine triphosphate, calcitonin gene-related peptide, and substance P [16,17]. Additionally, studies show that intravesical BoNT-A injection can reduce mast cell activity in the urothelium, suggesting an anti-inflammatory effect in the bladder [18]. The bladder afferent input modulation and anti-inflammatory effect play important roles in the mechanism of BoNT-A in some sensory problems, predominantly bladder disorders such as IC/BPS. The abovementioned mechanisms occurred in the bladder lamina propria and detrusor muscle; hence, BoNT-A must cross the urothelium to act on sub-urothelial tissue.

## 3. Liposomal BoNT-A for Functional Bladder Disorders

Liposomes are self-assembling spherical vesicles with at least one lipid bilayer. They have an aqueous solution core surrounded by a hydrophobic membrane; hence, hydrophilic solutes can be dissolved in the core, and the outer lipid bilayer can fuse with other lipid bilayer structures such as the cell membrane [19]. This structure was first described in the 1960s. It was widely used as a drug delivery vehicle to load nanoparticles for the administration of nutrients and drugs to cells [19]. Since 2004, Tyagi et al. have used liposome-encapsulated capsaicin intravesical administration in rats, and this revealed completely blocked micturition reflexes after administration [20]. A significant reduction of calcitonin gene-related peptide staining of afferent nerves in sub-urothelial tissue was also noted [20]. In 2009, Chuang et al. further administered liposome-encapsulated BoNT-A into rat bladders after acetic acid administration [21]. The results revealed that liposome-encapsulated BoNT-A can protect rat bladders from acetic acid injury by alleviating bladder inflammation and preserving the inter-contraction interval in urodynamic studies. More importantly, SNAP-25 expression decreased in bladders treated with liposome-encapsulated BoNT-A, proving that liposome can carry BoNT-A across uroepithelial cells and act on sub-urothelial nerve endings.

Intravesical injection of BoNT-A can relieve urinary frequency and incontinence in patients with OAB [11], and intravesical administration of liposome-encapsulated BoNT-A has similar therapeutic effects [22,23]. In our pilot randomized placebo control study [23], OAB patients were treated with a single intravesical administration of 200 U liposome-encapsulated BoNT-A or normal saline. In one month after the treatment, the urinary frequency and urgency episodes were significantly decreased in the liposome-encapsulated BoNT-A treated group, but not in the normal saline group. The following multicenter, double-blind and randomized study further confirmed the improvement of urinary frequency and urgency episodes, and urgency severity scores were significantly greater in the liposome-encapsulated BoNT-A administration group than that in placebo group [22]. The therapeutic effect could last for 3 months in the study, without any treatment related adverse event. It is worth noting that current studies on using liposome-encapsulated BoNT-A to treat patients with neurogenic detrusor overactivity are still lacking. 

Intravesical injection of BoNT-A could also improve bladder pain and urinary frequency in patients with IC/BPS [12]. We previously conducted a randomized placebo study to treat IC/BPS patients with intravesical administration of liposome-encapsulated BoNT-A 200U, BoNT-A 200U with normal saline, or normal saline alone [24]. Although the bladder pain improved in the liposome-encapsulated BoNT-A group, similar improvement was also observed in the other two groups. Liposome-encapsulated BoNT-A failed to demonstrate significant benefit compared to BoNT-A with normal saline or placebo, which was likely to be due to a significant placebo effect. In summary, previous studies revealed the liposome could be used as a vehicle to deliver BoNT-A cross uroepithelium and acted on the sub-urothelial nerve endings. Pilot studies also show that intravesical administration of liposome-encapsulated BoNT-A can improve lower urinary tract symptoms in patients with functional bladder disorders. However, further studies with a greater variety of patients are still necessary to confirm the clinical efficacy and to expand the applications.

## 4. TC-3 Hydrogel Embedding BoNT-A for Functional Bladder Disorders

Hydrogel products constitute a three-dimensional network of hydrophilic polymers, which are highly absorbent and hold a large amount of water while maintaining their structure [25]. Hydrogels allow for the encapsulation of drugs and provide an environment similar to animal tissues. They have been used as effective drug delivery systems [25]. In the urinary bladder, using intra-vesically-administered hydrogel for drug delivery can prolong the residence time in the bladder, sustain drug release, maintain drug concentration, and result in better therapeutic efficacy [26]. Hydrogels which contain cationic materials can increase drug penetration in the bladder wall because of the electrostatic interaction between the positively-charged cationic materials and the negatively-charged glycosaminoglycan layer in the uroepithelium [26]. Electrostatic interaction can rearrange the tight junctions between the uroepithelium cells, enhance paracellular permeability, and transport drugs into sub-urothelial tissue [26]. In recent years, intravesical administration of hydrogel- contained drugs, such as mitomycin C and doxorubicin, has been used to treat patients with bladder cancer. A newly developed commercial hydrogel TC-3 which shows excellent biocompatibility, prolonged drug release, and improved bladder mucosa adhesion has entered clinical trials for patients with bladder cancer [26].

TC-3 hydrogel embedded BoNT-A has also been used to treat patients with functional bladder disorders. Krhut et al. conducted a double-blind randomized study to assigned OAB patients to receive intravesical administration of normal saline, TC-3 hydrogel embedded BoNT-A (200 U), and TC-3 hydrogel embedded BoNT-A (200 U) with dimethyl sulfoxide or dimethyl sulfoxide only [27]. The OAB patients who received TC-3 hydrogel embedded BoNT-A (200 U) experienced better improvement regarding urinary leak, grade 3 or 4 urgency, and Patient Perception of Bladder Condition total score when compared to the other three groups. The post-void residual volume was not significantly increased in all groups, and no serious adverse events were observed. For IC/BPS, Rappaport et al. conducted a single-arm pilot study to treat patients with intravesical administration of TC-3 hydrogel embedded BoNT-A (200 U) [28]. Bladder pain and urinary frequency significantly decreased at 12 weeks follow up, despite the lack of a placebo control. Although preliminary studies revealed promising therapeutic outcomes, only two clinical trials with limited patients have been conducted.

It is worth noting that urinary tract infection developed in 1 of 9 (11.1%) [27] and 2 of 16 (12.5%) [28] patients in the two studies. Laboratory evidence revealed that liposome can deliver BoNT-A into sub-urothelial tissue [20,21]; however, such evidence is still lacking in TC-3 hydrogel embedded BoNT-A studies.

## 5. Low Energy Shock Wave to Promote Intravesical BoNT-A Delivery to Urothelium

Energy shock wave (ESW) is a type of stress wave that can carry energy and propagate through a medium [29]. Previous studies have revealed that using low ESW (LESW) in a chronically-injured tissue can promote neovascularization, regeneration, and reduce inflammation [30]. LESW is now widely used in treating patients with chronic joint pain, inflammation, and adhesion [29,31]. A previous animal study showed that LESW can reduce bladder pain, urinary frequency, and inflammation in rats with cyclophosphamide-induced bladder inflammation [32]. We recently performed a prospective, randomized, and placebo controlled study to evaluate the efficacy of LESW in treating patients with IC/BPS [33]. IC/BPS patients were assigned to LESW once a week for four weeks at the suprapubic bladder or placebo. Both study and control groups had statistically significant reductions on the visual analog scale pain score, but there was no significant difference between the two groups. In the LESW group, only two patients reported mild suprapubic pain during the procedure. No patient had urinary incontinence, retention, or infection in either groups.

ESW is also well known for its ability to alter cell membrane permeability [34]. Over the past 30 years, laboratory data have showed that underwater ESW is a safe, non-invasive method for molecular delivery into mammalian cells, and it has been used in numerous cell genetic modification studies [34]. Previous in vitro studies proved that using ESW can promote macromolecular drug delivery into cells [35]. Recently, a rat study showed that ESW can mediate local anesthetic drug transdermal delivery into caudal nerves [36]. In the urinary bladder, magnetic resonance imaging with contrast medium Gd-diethylenetriamine penta-acetic acid revealed increased rat urothelium permeability after ESW [37]. Also, rat studies revealed that intravesical administration of BoNT-A after ESW can suppress the acetic acid-induced bladder hyperactivity and inflammatory reaction [37]. Immunochemical staining also revealed decreased SANP-23 and SNAP-25 in rats with intravesical administration of BoNT-A after ESW [37]. The abovementioned evidence revealed that suprapubic ESW can promote intravesical BoNT-A delivery into urothelium, and potentially has clinical use.

We herein report a pilot clinical case series that explores the clinical efficacy of using suprapubic ESW to promote intravesical BoNT-A delivery in patients with IC/BPS. Six patients with refractory IC/BPS initially underwent cystoscopic hydro-distention and bladder biopsy to confirm their diagnosis. They then received intravesical administration of BoNT-A [200 U] (BOTOX^®^ Allergan, Irvine, CA) after emptying the bladder, followed by ESW. Patients were asked to hold their urine for at least one hour. The treatment was weekly and repeated for a total of four weeks. The ESW procedure was performed as in our previous study [33]. In summary, the shock wave probe was placed on the transmission gel over the suprapubic region above the urinary bladder, with 3000 shocks (3 pulses/second, and maximum energy flow density 0.25 mJ/mm). Treatment outcome was evaluated at one month after the fourth procedure, and patients again underwent cystoscopic hydro-distention with bladder biopsy. Bladder specimens were analyzed with immunochemical staining for cleaved SNAP25 (antibody brand: Gene Tex, GTX39119).

**Figure 1:**
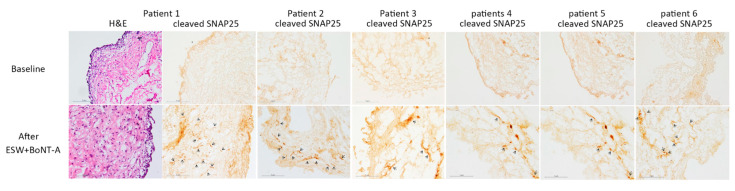
The bladder biopsies figures for H&E (hematoxylin and eosin stain) and immunochemical staining for cleaved SNAP-25 in patients with IC/BPS. Immunoreactivity of cleaved SNAP-25 was detected in all of the six bladder specimens after intravesical installation BoNT-A with ESW.

All patients tolerated the procedure well without any complications; however, the therapeutic effect was not significant. Reduction of pain on the visual analog scale, and symptoms score in Interstitial Cystitis Symptom Index and Problem Index, were not significantly improved in this pilot study with six patients (Table 1). Two of the six (33%) patients had global response assessment ≥ 2, and the other four patients (67%) had global response assessment = 1. In the immunochemical staining, cleaved SNAP25 was detected in all of the bladder specimens after BoNT-A administration with ESW (Figure 1). Our results revealed that ESW can promote passage of BoNT-A across the uroepithelium, and mediate BoNT-A to work on the bladder nerve endings. Although the clinical efficacy was not significant in our current preliminary study, using ESW to promote intravesical BoNT-A delivery is still reasonable and promising. Further studies with different ESW protocols or an increased BoNT-A dose may improve treatment outcome.

We summarized current clinical evidence of non-invasive intravesical BoNT-A delivery for patients with functional bladder disorders in the Table 2. Using vehicles to intra-vesically deliver BoNT-A for functional bladder disorders is promising, but still requires further study to prove clinical efficacy.

## 6. Conclusions

Using vehicles to intra-vesically deliver BoNT-A for functional bladder disorders is promising. Further studies are necessary to confirm the efficacy and explore novel applications.

## Figures and Tables

**Table 1:** The clinical parameters in patients with IC/BPS at baseline and 1 month after intravesical BoNT-A installation with ESW.

	Baseline (*n* = 6)	1 Month after Treatment (*n* = 6)	*p*-Value
ICSI	9.5 ± 4.0	9.2 ± 2.6	0.777
ICPI	10.2 ± 3.3	7.8 ± 1.5	0.065
VAS	3.2 ± 1.0	2.7 ± 1.8	0.580
FBC (mL)	308.3 ± 97.0	320.0 ± 102.0	0.220
Qmax (mL/s)	16.3 ± 7.7	11.0 ± 5.6	0.017
Vol (mL)	384.2 ± 116.6	270.8 ± 140.5	0.020
PVR (mL)	13.2 ± 14.5	13.3 ± 21.6	0.989
Cleaved SNAP-25	0%	100%	
GRA	0	1.17 ± 1.16	

ICSI and ICPI: Interstitial Cystitis Symptom Index and Problem Index; VAS: visual analog scale for pain; FBC: functional bladder capacity in urodynamic study; Qmax: maximal flow rate: Vol: voided volume; PVR: post-voiding residual volume; SNAP-25: synaptosome-associated protein 25; GRA: global response assessment.

**Table 2:** Current clinical evidence of non-invasive intra-vesical BoNT-A delivery for patients with functional bladder disorders.

		OAB		IC/BPS		
	Study Design	Authors, Year	Outcome	Study Design	Authors, Year	Outcome
liposome	Placebo controlled RCT	Chuang YC, 2014	significant decrease in urgency severity scores compared to placebo [22]	Placebo controlled RCT	Chuang, YC, 2017	Improved bladder pain but not superior to placebo [24]
		Kuo HC, 2014	significant reduction of urinary frequency and urgency in Lipotoxin group in compared to control group [23]			
TC-3 gel	Placebo controlled RCT	Krhut J, 2016	Improved more urinary leak and grade 3 or 4 urgency than placebo [27].	Single-arm prospective cohort study	Rappaport, YH, 2018	bladder pain and urinary frequency significantly decreased [28]
ESW		N/A		Case series	Kuo HC, 2021	Bladder symptoms not significantly improved, but IHC showed ESW could promote BoNT-A intravesical delivery

OAB: overactive bladder; IC/BPS: interstitial cystitis/bladder pain syndrome; RCT: randomized controlled trial; ESW: energy shock wave; IHC: immunochemical staining.

## Data Availability

Not applicable.

## References

[1] Irwin D.E., Milsom I., Hunskaar S., Reilly K., Kopp Z., Herschorn S., Coyne K., Kelleher C., Hampel C., Artibani W. (2006). Population-based survey of urinary incontinence, overactive bladder, and other lower urinary tract symptoms in five countries: Results of the EPIC study. Eur. Urol..

[2] Irwin D.E., Kopp Z.S., Agatep B., Milsom I., Abrams P. (2011). Worldwide prevalence estimates of lower urinary tract symptoms, overactive bladder, urinary incontinence and bladder outlet obstruction. BJU Int..

[3] Suskind A.M., Berry S.H., Ewing B.A., Elliott M.N., Suttorp M.J., Clemens J.Q. (2013). The prevalence and overlap of interstitial cystitis/bladder pain syndrome and chronic prostatitis/chronic pelvic pain syndrome in men: Results of the RAND Interstitial Cystitis Epidemiology male study. J. Urol..

[4] Tay K., Khan A. (2017). Patient Satisfaction on Overactive Bladder Treatment. Curr. Bladder Dysfunct. Rep..

[5] Hanno P.M., Erickson D., Moldwin R., Faraday M.M., American Urological A. (2015). Diagnosis and treatment of interstitial cystitis/bladder pain syndrome: AUA guideline amendment. J. Urol..

[6] De Seze M., Wiart L., de Seze M.P., Soyeur L., Dosque J.P., Blajezewski S., Moore N., Brochet B., Mazaux J.M., Barat M. (2004). Intravesical capsaicin versus resiniferatoxin for the treatment of detrusor hyperreflexia in spinal cord injured patients: A double-blind, randomized, controlled study. J. Urol..

[7] Hung M.J., Tsai C.P., Lin Y.H., Huang W.C., Chen G.D., Shen P.S. (2019). Hyaluronic acid improves pain symptoms more than bladder storage symptoms in women with interstitial cystitis. Taiwan J. Obstet. Gynecol..

[8] Liu S., Zhang C., Peng L., Lu Y., Luo D. (2020). Comparative effectiveness and safety of intravesical instillation treatment of interstitial cystitis/bladder pain syndrome: A systematic review and network meta-analysis of randomized controlled trials. Int. Urogynecol. J..

[9] Nigam P.K., Nigam A. (2010). Botulinum toxin. Indian J. Dermatol..

[10] Dykstra D.D., Sidi A.A., Scott A.B., Pagel J.M., Goldish G.D. (1988). Effects of botulinum A toxin on detrusor-sphincter dyssynergia in spinal cord injury patients. J. Urol..

[11] Anger J.T., Weinberg A., Suttorp M.J., Litwin M.S., Shekelle P.G. (2010). Outcomes of intravesical botulinum toxin for idiopathic overactive bladder symptoms: A systematic review of the literature. J. Urol..

[12] Kuo H.C., Jiang Y.H., Tsai Y.C., Kuo Y.C. (2016). Intravesical botulinum toxin-A injections reduce bladder pain of interstitial cystitis/bladder pain syndrome refractory to conventional treatment-A prospective, multicenter, randomized, double-blind, placebo-controlled clinical trial. Neurourol. Urodyn..

[13] Lightner D.J., Gomelsky A., Souter L., Vasavada S.P. (2019). Diagnosis and Treatment of Overactive Bladder (Non-Neurogenic) in Adults: AUA/SUFU Guideline Amendment 2019. J. Urol..

[14] Birder L.A., de Groat W.C. (2007). Mechanisms of disease: Involvement of the urothelium in bladder dysfunction. Nat. Clin. Pract. Urol..

[15] Solabre Valois L., Wilkinson K.A., Nakamura Y., Henley J.M. (2020). Endocytosis, trafficking and exocytosis of intact full-length botulinum neurotoxin type a in cultured rat neurons. Neurotoxicology.

[16] Aoki K.R. (2003). Evidence for antinociceptive activity of botulinum toxin type A in pain management. Headache.

[17] Kaya S., Hermans L., Willems T., Roussel N., Meeus M. (2013). Central sensitization in urogynecological chronic pelvic pain: A systematic literature review. Pain Physician.

[18] Shie J.H., Liu H.T., Wang Y.S., Kuo H.C. (2013). Immunohistochemical evidence suggests repeated intravesical application of botulinum toxin A injections may improve treatment efficacy of interstitial cystitis/bladder pain syndrome. BJU Int..

[19] Alavi M., Karimi N., Safaei M. (2017). Application of Various Types of Liposomes in Drug Delivery Systems. Adv. Pharm. Bull..

[20] Tyagi P., Chancellor M.B., Li Z., De Groat W.C., Yoshimura N., Fraser M.O., Huang L. (2004). Urodynamic and immunohistochemical evaluation of intravesical capsaicin delivery using thermosensitive hydrogel and liposomes. J. Urol..

[21] Chuang Y.C., Tyagi P., Huang C.C., Yoshimura N., Wu M., Kaufman J., Chancellor M.B. (2009). Urodynamic and immunohistochemical evaluation of intravesical botulinum toxin A delivery using liposomes. J. Urol..

[22] Chuang Y.C., Kaufmann J.H., Chancellor D.D., Chancellor M.B., Kuo H.C. (2014). Bladder instillation of liposome encapsulated onabotulinumtoxina improves overactive bladder symptoms: A prospective, multicenter, double-blind, randomized trial. J. Urol..

[23] Kuo H.C., Liu H.T., Chuang Y.C., Birder L.A., Chancellor M.B. (2014). Pilot study of liposome-encapsulated onabotulinumtoxina for patients with overactive bladder: A single-center study. Eur. Urol..

[24] Chuang Y.C., Kuo H.C. (2017). A Prospective, Multicenter, Double-Blind, Randomized Trial of Bladder Instillation of Liposome Formulation OnabotulinumtoxinA for Interstitial Cystitis/Bladder Pain Syndrome. J. Urol..

[25] Kopecek J. (2007). Hydrogel biomaterials: A smart future?. Biomaterials.

[26] Qiu H., Guo H., Li D., Hou Y., Kuang T., Ding J. (2020). Intravesical Hydrogels as Drug Reservoirs. Trends Biotechnol..

[27] Krhut J., Navratilova M., Sykora R., Jurakova M., Gartner M., Mika D., Pavliska L., Zvara P. (2016). Intravesical instillation of onabotulinum toxin A embedded in inert hydrogel in the treatment of idiopathic overactive bladder: A double-blind randomized pilot study. Scand. J. Urol..

[28] Rappaport Y.H., Zisman A., Jeshurun-Gutshtat M., Gerassi T., Hakim G., Vinshtok Y., Stav K. (2018). Safety and Feasibility of Intravesical Instillation of Botulinum Toxin-A in Hydrogel-based Slow-release Delivery System in Patients With Interstitial Cystitis-Bladder Pain Syndrome: A Pilot Study. Urology.

[29] Modena D.A.O., da Silva C.N., Grecco C., Guidi R.M., Moreira R.G., Coelho A.A., Sant’Ana E., de Souza J.R. (2017). Extracorporeal shockwave: Mechanisms of action and physiological aspects for cellulite, body shaping, and localized fat-Systematic review. J. Cosmet. Laser Ther..

[30] Liu T., Shindel A.W., Lin G., Lue T.F. (2019). Cellular signaling pathways modulated by low-intensity extracorporeal shock wave therapy. Int. J. Impot. Res..

[31] Mariotto S., de Prati A.C., Cavalieri E., Amelio E., Marlinghaus E., Suzuki H. (2009). Extracorporeal shock wave therapy in inflammatory diseases: Molecular mechanism that triggers anti-inflammatory action. Curr. Med. Chem..

[32] Wang H.J., Lee W.C., Tyagi P., Huang C.C., Chuang Y.C. (2017). Effects of low energy shock wave therapy on inflammatory moleculars, bladder pain, and bladder function in a rat cystitis model. Neurourol. Urodyn..

[33] Chuang Y.C., Meng E., Chancellor M., Kuo H.C. (2020). Pain reduction realized with extracorporeal shock wave therapy for the treatment of symptoms associated with interstitial cystitis/bladder pain syndrome—A prospective, multicenter, randomized, double-blind, placebo-controlled study. Neurourol. Urodyn..

[34] Datey A., Chakravortty D., Gopalan J. (2018). An overview of a novel use of shockwaves to alter cell permeability: Comment on “Shock wave-induced permeabilization of mammalian cells” by Luz M. Lopez-Marin et al. Phys. Life Rev..

[35] Battula N., Menezes V., Hosseini H. (2016). A miniature shock wave driven micro-jet injector for needle-free vaccine/drug delivery. Biotechnol. Bioeng..

[36] Luh J.J., Huang W.T., Lin K.H., Huang Y.Y., Kuo P.L., Chen W.S. (2018). Effects of Extracorporeal Shock Wave-Mediated Transdermal Local Anesthetic Drug Delivery on Rat Caudal Nerves. Ultrasound Med. Biol..

[37] Chuang Y.C., Huang T.L., Tyagi P., Huang C.C. (2016). Urodynamic and Immunohistochemical Evaluation of Intravesical Botulinum Toxin A Delivery Using Low Energy Shock Waves. J. Urol..

